# Global progress in clinical research on human respiratory syncytial virus vaccines

**DOI:** 10.3389/fmicb.2024.1457703

**Published:** 2024-09-02

**Authors:** Ruofan Peng, Chenghao Chen, Qian Chen, Yuwen Zhang, Renjin Huang, Yanjun Zhang, Jianhua Li

**Affiliations:** ^1^School of Medical Technology and Information Engineering, Zhejiang Chinese Medical University, Hangzhou, China; ^2^School of Public Health, Hangzhou Medical College, Hangzhou, China; ^3^School of Laboratory Medicine and Life Science, Wenzhou Medical University, Wenzhou, China; ^4^Key Laboratory of Public Health Detection and Etiological Research of Zhejiang Province, Department of Microbiology, Zhejiang Provincial Center for Disease Control and Prevention, Hangzhou, China

**Keywords:** human respiratory syncytial virus, lower respiratory tract disease, vaccine development, clinical research, immune population

## Abstract

Human respiratory syncytial virus (hRSV) not only affects newborns but also older adults, contributing to a substantial worldwide burden of disease. However, only three approved hRSV vaccines remain commercially available to date. The development of a safe, practical and broad-spectrum vaccine suitable for all age groups remains extremely challenging. Using five different approaches—live-attenuated, recombinant-vector, subunit, particle-based, and mRNA—nearly 30 hRSV vaccine candidates are currently conducting clinical trials worldwide; moreover, > 30 vaccines are under preclinical evaluation. This review presents a comprehensive overview of these hRSV vaccines along with prospects for the development of infectious disease vaccines in the post-COVID-19 pandemic era.

## 1 Introduction

Human respiratory syncytial virus (hRSV) is a primary etiological agent underlying acute lower respiratory infections (ALRIs) in newborns, young children, older adults and immune-compromised individuals all over the globe (GBD 2016 Lower Respiratory Infections Collaborators, 2018; Shi et al., 2017). hRSV infections can present with a wide range of clinical manifestations, from moderate respiratory infections to severe lower respiratory tract diseases (LRTDs) such as bronchiolitis or pneumonia, with severe infections even affecting organs other than the respiratory system. Approximately 90% of children aged < 2 years are infected with hRSV and susceptible to repeated infections, which poses a serious threat to their health (Glezen et al., 1986). Over the years after the end of the COVID-19 pandemic, the incidence of hRSV increased owing to the relaxation of public health measures. The surveillance data from the Respiratory Syncytial Virus Global Epidemiology Network (RSV GEN) indicated that 33 million hRSV-related ALRI cases were reported worldwide in 2019, which included 3.6 million hospitalizations and 26,300 deaths. Approximately 6.6 million ALRI cases of these cases were infants aged 0–6 months, which led to 1,400 hospitalizations and 13,300 deaths. More than 95% of ALRI cases and > 97% of hRSV-related deaths were reported in low- or middle-income countries (Li et al., 2022). Older adults aged ≥ 65 years, particularly those with an underlying respiratory or cardiac disorder or with a congenital immunodeficiency or compromised immune system, are more prone to severe hRSV infections, which may even result in death. Epidemiological studies have demonstrated that hRSV infection prevalence in older adults ranges from 2 to 10%. With the global population continuing to age, hRSV disease burden among older adults is expected to exceed 30% over 2020–2025. Consequently, safe and effective hRSV vaccines, which afford protection across several age groups, are highly required to meet global demand, especially in low- and middle-income countries.

## 2 Overview of hRSV

**FIGURE 1:**
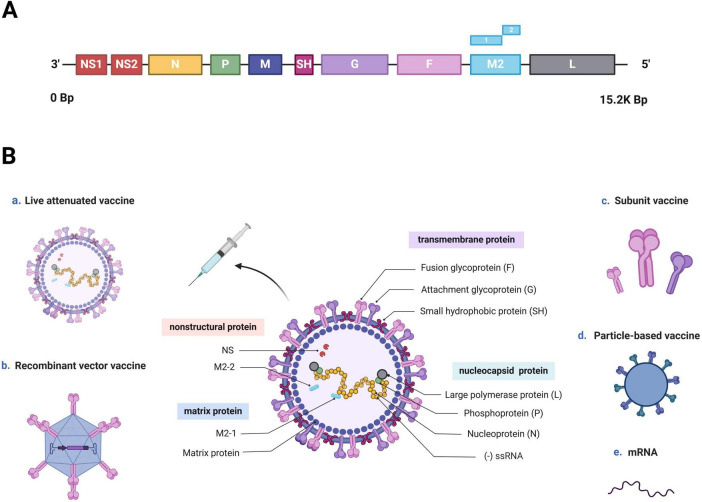
hRSV genome, structure, and vaccine development approaches (Created with BioRender.com). **(A)** hRSV genome sequence. The entire hRSV genome is 15.2 kb long and contains 10 genes encoding 11 proteins. **(B)** Schematic of an hRSV virion, and various approaches used for vaccine development. hRSV is an enveloped virus with a nonsegmented, negative-sense RNA genome (-ssRNA). It is composed of eight structural and three nonstructural proteins. The nonstructural proteins are NS1, NS2, and the RNA regulatory factor M2-2, whereas the structural proteins include three transmembrane proteins (F and G proteins and SH), three nucleocapsid proteins (large polymerase protein L, phosphoprotein P, and nucleoprotein N) and two matrix proteins (matrix protein M and transcription antitermination factor M2-1). Recent hRSV vaccine development has been based on five mechanisms: live-attenuated, recombinant-vector, subunit, particle-based, and mRNA.

hRSV is an enveloped, negative-sense RNA virus, which is a member of the *Orthopneumovirus* genus and *Pneumoviridae* family (Afonso et al., 2016; Rima et al., 2017). The full hRSV genome is 15.2 kb long and encodes 11 proteins, including 8 structural and 3 nonstructural proteins. The nonstructural proteins include NS1, NS2, and M2-2, whereas the structural proteins comprise three transmembrane proteins (G, F, and SH), two matrix proteins (M and M2-1) and three nucleocapsid proteins (L, N, and P) (Figure 1A; Mejias et al., 2020; McLellan et al., 2013b). Wherein, fusion glycoprotein (F protein) and attachment glycoprotein (G protein) are the main targets of neutralizing antibodies (nAbs). The F protein have been consequently considered crucial targets in recent vaccine research due to its conserved and highly neutralization sensitive epitopes (Anderson et al., 1988; Walsh and Hruska, 1983). This allows the nAb induced by the F protein to crossprotect against varied hRSV strains (Jares Baglivo and Polack, 2019; DeFord et al., 2019). G proteins are type-specific: different hRSV strains demonstrate variations in their G protein sequences. Consequently, hRSV strains are classified into subtypes A and B (Afonso et al., 2016; Broadbent et al., 2015). In a systematic study of the hRSV global epidemiology, subtype A demonstrates a higher prevalence accounting for 60% of reported hRSV infections worldwide—59.6% in the Northern Hemisphere and 61.9% in the Southern Hemisphere. However, over the past decade, a larger proportion of subtype B infections has been observed across all continents, except Asia. Both subtype A and B strains are typically cotransmissible, and they cause epidemics alternately over 1–2-year cycles (Cantú-Flores et al., 2022).

## 3 The major targets of hRSV neutralizing antibodies

### 3.1 F protein

**FIGURE 2:**
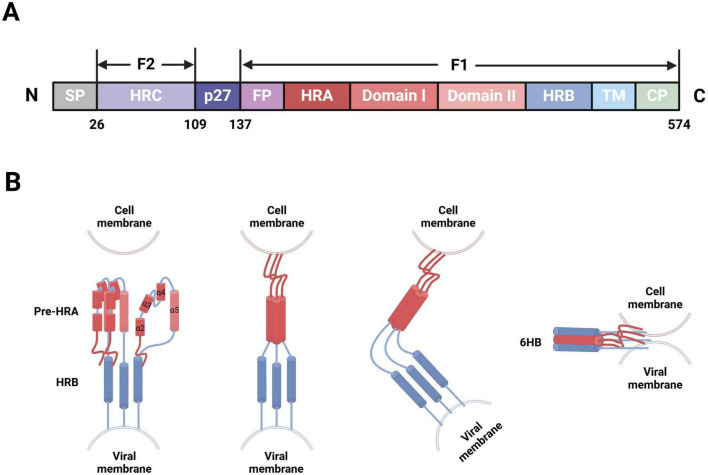
hRSV F protein-membrane fusion model (Created with BioRender.com). **(A)** Schematic of the modular structure of hRSV F protein, depicting the N-terminus, signal peptide (SP), heptad repeat C (HRC), p27 peptide, fusion peptide (FP), heptad repeat A (HRA), functional domain I/II, heptad repeat B (HRB), transmembrane region (TM), and C-terminus. **(B)** After the FP at the N-terminus of F1, four short α-helices are joined by three nonhelical peptides. When triggered, these peptides refold into α-helices, forming a long single HRA α-helix (red) that propels FP into the target cell membrane. Then, the molecule folds in half, long HRA α-helices trimerize, and HRB α-helices (blue) become inserted into the grooves between the HRA units to form a stable six-helix bundle (6HB). Consequently, membrane fusion initiates through viral-cell membrane fusion.

F protein is a type I transmembrane glycoprotein, initially expressed as an inactive precursor protein, F0, consisting of 574 amino acids (Martin-Gallardo et al., 1989). F0 becomes activated through proteolytic cleavage by cellular furin-like proteinases, which results in the formation of a functional fusion glycoprotein. This process generates several fragments in the following sequential order from the N- to C-terminus: signal peptide (aa2-20), signal peptide cleavage site (aa21-25), F2 subunit (aa26-109), p27 peptide (aa110-136), and F1 subunit (aa137-574). The F1 and F2 subunits subsequently form heterodimers through disulfide bonds; three of these heterodimers form a mature F protein trimer (Anderson et al., 1992; Sugrue et al., 2001). The F1 subunit contains multiple domains, including a fusion peptide (FP), heptad repeat A (HRA), functional domain I/II, heptad repeat B (HRB), transmembrane protein (TM), and cytoplasmic tail region (CP). Notably, the FP is located at the hydrophobic N-terminus of F1 (aa137-155) and is embedded within the trimeric cavity. With both adhesion and fusion functionalities, the F protein facilitates viral particle entry into cells and syncytia formation. The hydrophobic N-terminus of F1 plays a crucial mediating role in viral particle-target cell membrane fusion. In contrast, the F2 subunit, including heptad repeat C (HRC), is the sole determinant of host cell specificity during hRSV infection (Figure 2A; McLellan et al., 2010; Swanson et al., 2011; Yunus et al., 2010).

The infectious hRSV surface simultaneously presents two distinct conformations of F protein: the metastable prefusion form (PreF), which exists on viral particle membranes before virus-cell interaction, and the stable postfusion form (PostF), which forms after PreF refolds at viral-cell membrane fusion. The mechanism triggering this refolding process, however, remains unclear. Thus, the transition of F protein from PreF to PostF also signifies the fusion between viral and target cell membranes. After G protein binds to its receptors on the target cell membrane, the FP is released from the trimeric protein cavity, which leads to the refolding of the HRA secondary structure within the F1 subunit and the formation of a long α-helix structure, followed by trimerization. The FP then inserts into the adjacent target cell membrane, allowing HRB to bind to the groove within the HRA trimer, which results in the formation of a stable six-helix bundle structure (Zhao et al., 2000). F protein folds at the center, spanning both the target cell and viral particle membranes, thereby completing the fusion of the viral envelope and cell membrane (Figure 2B; Battles and McLellan, 2019). Recent advancements in cryoelectron microscopy technology have allowed researchers to observe both PreF and PostF structures on viral particle surfaces (Liljeroos et al., 2013), validating the presence of the metastable PreF and its eventual transition to the stable PostF. However, conditions such as high temperature and low osmolarity can induce conformational changes in PreF (Yunus et al., 2010; Chaiwatpongsakorn et al., 2011).

**FIGURE 3:**
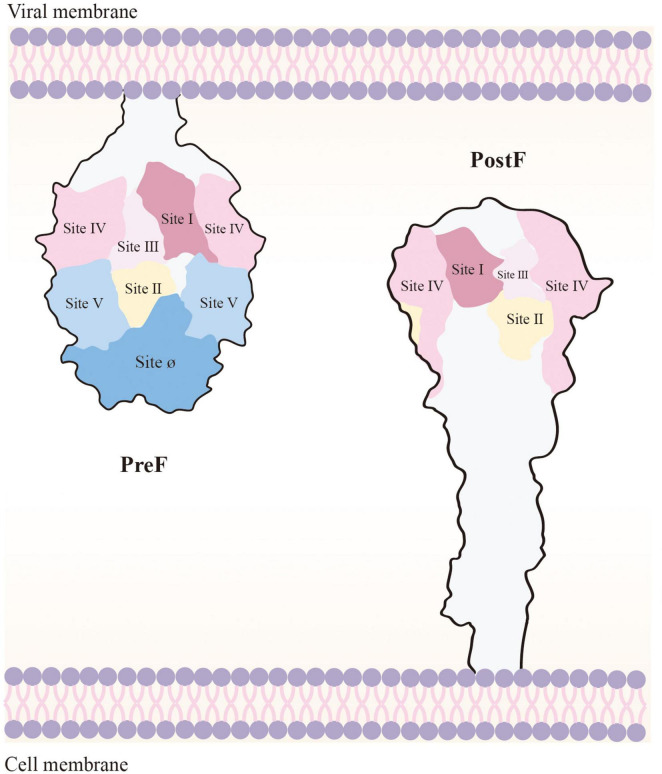
Loss of Ø and V, the PreF-specific sites, in the transition to PostF. As the membrane of the virus and the host cell merge, PostF retains antigenic sites I to IV; however, this leads to loss of Ø and V (i.e., the targets of most potent RSV-nAbs). PostF stem is composed of a six-helix bundle comprising the long α-helices from refolded V and Ø and the α-helical stem of PreF. Removal of the transmembrane domain of PreF, which enables refolding in the absence of membranes, results in PostF production.

F protein surface bears various neutralizing epitopes, including sites Ø (McLellan et al., 2013a), I (Anderson et al., 1985), II (Lopez et al., 1993), III (Corti et al., 2013), IV (Wu et al., 2007), V, VI (Lopez et al., 1998), and VIII (Mousa et al., 2017). PreF and PostF demonstrate sites I, II, III, IV, and VI. In contrast, sites Ø and V are exclusive to PreF, and they induce relatively elicit nAb production. Site Ø, located at the top of PreF, is particularly crucial, and maintaining its stability is vital to vaccine development (Figure 3; Ngwuta et al., 2015).

### 3.2 G protein

G protein is a polypeptide precursor, approximately 300 amino acids in length. It possesses a single hydrophobic structural domain (aa38-63) near the N-terminus, which serves as a cosignaling and membrane anchoring structural domain (Wertz et al., 1985). In contrast to other paramyxovirus G proteins, it does not perform hemagglutination or neuraminidase functions. The central conserved region of G protein (aa163-189) contains a segment of 13 amino acids (aa164-176). It also includes four cysteines highly conserved across all hRSV strains; they form disulfide bonds between Cys173 and Cys186 and between Cys176 and Cys182, which results in the formation of a partially folded cysteine lasso motif (Langedijk et al., 1996). The remaining sequence of G protein exhibits group specificity. Moreover, the central conserved region of G protein contains a CX_3_C motif (aa182-186) that can bind to CX_3_CR1—the specific receptor for the chemokine fractalkine in lung ciliated epithelial cells—and induce leukocyte chemotaxis (Tripp et al., 2001). In a study, the inoculation of mice with IgG induced by peptides from the central conserved region of G protein blocked G-CX_3_CR1 interactions, thus ameliorating hRSV infection (Bergeron et al., 2021).

The primary function of G protein is to act as an adhesion agent, facilitating the binding of the virus to the target cell through interactions with cell surface molecules. Consequently, it also acts as an immunogen for vaccine development, stimulating nAb production. During the cellular replication cycle of hRSV, approximately one-sixth of G protein is secreted from the host cell as sG, a soluble secretory protein, whereas the remaining G protein mG is localized to the membrane and subsequently transported to the surface of the viral membrane during viral exocytosis. Most of the neutralizing antigenic epitopes are present in mG (Bukreyev et al., 2008). The antigenic epitopes on the G protein surface can be divided into three types: (i) a conserved epitope in all strains, located within the conserved 13 amino acid region of the unglycosylated central region; (ii) a group-specific epitope, which partially overlaps with the conserved epitope but is specific to strains within the same antigenic group; and (iii) an epitope specific to selected strains within the same antigenic group, which is located in the C-terminal hypervariable region of the extracellular domain of G protein (Martínez et al., 1997). Vaccination targeting G proteins induces a Th2-biased immune response (Acosta et al., 2015), and the presence of G proteins in vaccine candidates such as virus-like particles (VLPs) affects F protein conformations and the ensuing immune response to F proteins, resulting in a considerable increase in nAb titers and providing improved protection against hRSV infections (McGinnes Cullen et al., 2023).

### 3.3 Other proteins

Small hydrophobic (SH) protein is the smallest protein in hRSV membranes. Although it is not essential for hRSV replication, SH protein induces membrane permeability in liposomes. Moreover, it can form a pentameric ion channel structure, associated with viral evasion from host cell immunity (Araujo et al., 2016). Antibodies against SH protein do not possess neutralizing activity, but they can counteract viral infection through antibody-dependent cytotoxicity (Schepens et al., 2015; Schepens et al., 2014). hRSV-infected host cells demonstrate numerous detectable SH proteins, indicating that SH proteins are a potential target for hRSV vaccine development. Other eight hRSV proteins, which also play distinct roles in viral infection and immune system evasion, may provide valuable insights into hRSV vaccine design.

## 4 hRSV vaccine development history and challenges

**FIGURE 4:**
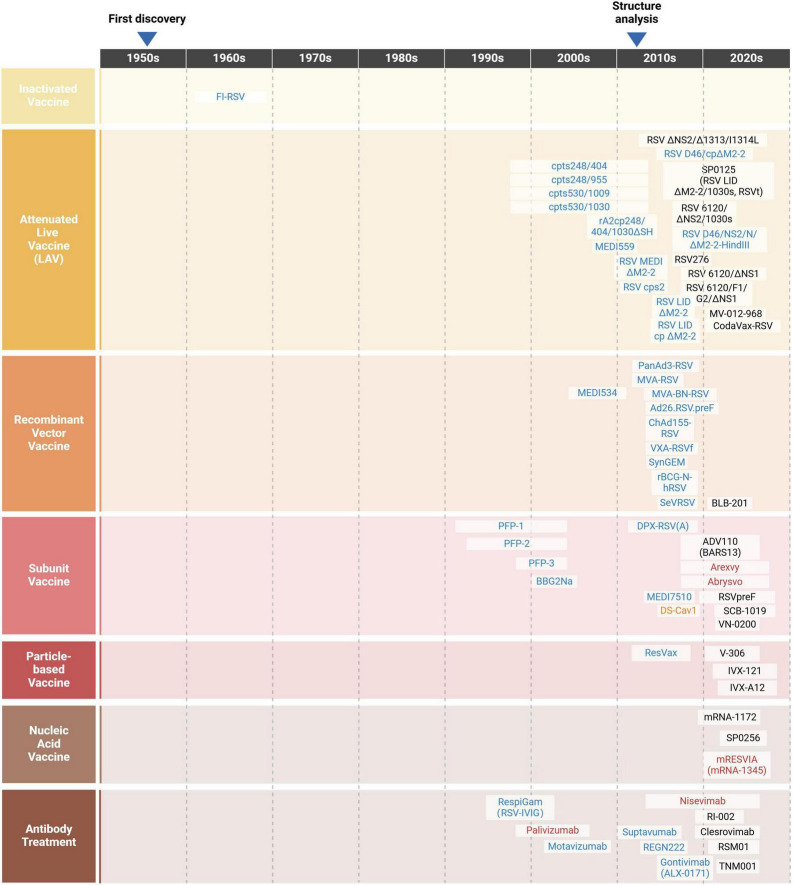
A timeline of the journey of hRSV (Created with BioRender.com). Following the initial isolation of hRSV from children with ALRI in 1957, studies on vaccines and antibodies were conducted. Unfortunately, these research stagnated during the 1970s and 1980s after the attempt of FI-RSV vaccine failed in 1969. In the 1990s, scientists delved deeply into the roles of hRSV surface glycoproteins, contributing significantly to the development of vaccines and antibody treatment strategies. In the early 2010s, the successful structural analysis of F protein led to a surge in vaccine and antibody candidates entering clinical trials. Currently, over 20 candidates are in the development stage (in black), with several failures or not under research now (in blue), one research tool (in orange) as well as three vaccines and two antibody treatments licensed for marketing (in red).

1957 saw the first isolation of hRSV from children with ALRI, which sparked research into the vaccine development (Figure 4). However, since the failure of the formalin-inactivated hRSV (FI-RSV) vaccine triggered enhanced respiratory disease (ERD) in the 1960s, progress in the development of preventive formulations against hRSV infections has remained slow (Kim et al., 1969). More than 20 attempts at vaccines have entered clinical trials within the last 60 years, but all have ultimately failed. Several obstacles and considerations, including safety, efficacy and immunological populations, limit the creation of hRSV vaccines (Table 1).

**TABLE 1:** The hRSV vaccines failed in clinical trials.

Vaccine	Type	Antigen	Target population	Cause of failure
FI-RSV	Inactivated vaccine	PostF protein	Infants	Insufficient security: the vaccine led to ERD
cpts248/955	LAV	All viral proteins	Infants	Insufficient security: the vaccine was not sufficiently attenuated
cpts248/404	LAV	All viral proteins	Infants	Insufficient security: the vaccine was not sufficiently attenuated
Cpts530/1030	LAV	All viral proteins	Infants	Insufficient security: the vaccine was not sufficiently attenuated
rA2cp248/404/1030ΔSH	LAV	All viral proteins except SH	Infants	Insufficient validity: the vaccine induced low levels of nAb titers
MEDI559	LAV	All viral proteins except SH	Infants	This vaccine was prone to revertant mutations
RSV cps2	LAV	All viral proteins except SH	Infants	Insufficient validity: both the vaccination group and the placebo group experienced the same frequency of fever and respiratory disease
RSV MEDI ΔM2-2	LAV	All viral proteins except M2-2	Infants	Insufficient validity: the peak titer of vaccine virus shed in nasal wash (NW) specimens was approximately 100-fold lower for MEDI ΔM2-2
RSV LID ΔM2-2	LAV	All viral proteins except M2-2	Infants	Insufficient security: the higher replication may make it poorly tolerated in some recipients when administered to large populations
RSV D46/NS2/N/ΔM2-2-HindIII	LAV	All viral proteins sexcept M2-2	Infants	Insufficient security: the vaccine was not sufficiently attenuated
MEDI-534	Recombinant vector vaccine	PostF protein	Infants	Insufficient security: the vaccine demonstrated weak immunogenicity and genomic instability
PanAd3-RSV	Recombinant vector vaccine	F, N, and M2-1 protein	Older adults	–
MVA-RSV	Recombinant vector vaccine	F, N, and M2-1 protein	Older adults	–
MVA-BN-RSV	Recombinant vector vaccine	F, G (subtype A/B), N, and M2 protein	Older adults	Insufficient validity: the vaccine failed to achieve the anticipated efficacy
Ad26.RSV.preF	Recombinant vector vaccine	PreF protein	Infants and older adults	–
ChAd155-RSV	Recombinant vector vaccine	F, N and M2-1 protein	Infants and children	Insufficient validity: the vaccine failed to achieve the anticipated efficacy
VXA-RSVf	Recombinant vector vaccine	F protein	–	–
SynGEM	Recombinant vector vaccine	PostF protein	Adults	Insufficient validity: the vaccine failed to induce the nAbs
rBCG-N-hRSV	Recombinant vector vaccine	N protein	Infants and older adults	–
SeVRSV	Recombinant vector vaccine	F protein	Infants	Insufficient validity: the vaccine failed to achieve the anticipated efficacy
ResVax	Particle-based vaccine	PostF protein	Infants, pregnant women and older adults	Insufficient validity: the vaccine failed to achieve the anticipated efficacy
BBG2Na	Subunit vaccine	G protein	Adults	Insufficient security: the vaccine caused type III hypersensitivity reactions
PFP	Subunit vaccine	Purified F protein	Infants and older adults	Insufficient validity: the vaccine demonstrated low yield and protection effect
MEDI7510	Subunit vaccine	PostF protein	Older adults	Insufficient validity: the vaccine induced low levels of nAb titers
DPX-RSV(A)	Subunit vaccine	SH protein	Older adults	–

–means not applicable or not available.

### 4.1 Safety concerns

Throughout the entire vaccine development process, safety is considered a primary concern. In 1969, Pfizer introduced FI-RSV, the first hRSV vaccine. Clinical testing was conducted on infants and younger children. However, within 9 months post-vaccination, those vaccinated with FI-RSV became reinfected with hRSV; approximately 80% of these infections resulted in severe illness and hospitalization, and two children died (Kim et al., 1969). Follow-up studies revealed that the failure of this vaccine was attributable to all F proteins on the virus surface being PostF. Consequently, the vaccine induced the production of low titers of nAbs or the production of numerous non-nAbs. This led to the formation of many immune complexes being deposited in the lung airways instead of being neutralized by hRSV during reinfection. Consequently, allergic inflammatory reactions, leading to ERD, occurred (Waris et al., 1996). In addition, the reinfection of cotton mice with hRSV after immunization with FI-RSV led to antibody-dependent enhancement (ADE) similar to that caused by dengue virus (van Erp et al., 2017). Thereafter, researchers continued analyzing the reasons for the undesirable results and proceeded with caution in hRSV vaccine development. However, after the failure of FI-RSV, the use of two vaccines was discontinued due to safety concerns. The first one was the G protein-based vaccine BBG2Na, which was discontinued because it could cause type III hypersensitivity reactions (Power et al., 2001). Moreover, MEDI-534, a bovine parainfluenza virus type 3-based vector vaccine, demonstrated weak immunogenicity and genomic instability in phase I clinical trials. As such, the vaccine’s further clinical trials are not permitted to proceed (Yang et al., 2013).

### 4.2 Insufficient validity

As F protein is unstable and prone to conformational transitions, hRSV vaccines were developed based on PostF design until McLellan optimally modified F protein to a stable state in 2013 (McLellan et al., 2013a). For instance, the subunit vaccine MEDI7510 demonstrated a favorable safety profile in phase I clinical trials conducted in individuals over 60 years of age; however, its further development was terminated due to insufficient effectiveness of antibodies induced by PostF (Falloon et al., 2016; Falloon et al., 2017). The novel recombinant vector vaccine SynGEM could induce systemic and mucosal immunity via nasal drop immunization in phase I clinical trials, which led to secretory immunoglobulin A (IgA) production intranasally; however, nAbs against site Ø were not detected in the participant, indicating that F protein in this vaccine was PostF (Ascough et al., 2019). The particulate vaccine ResVax, which had entered phase III clinical trials demonstrated a good safety and immunization effect in both preclinical and phase II clinical trials (Glenn et al., 2016; August et al., 2017). However, the vaccine demonstrated poor protection in a phase III clinical trial on 12,000 older adults aged ≥ 60 years; moreover, it failed to meet the primary endpoint in a phase III clinical trial on > 4,000 pregnant women, and the protection effect was poor in infants (Madhi et al., 2020). These results indicate that although PostF is more stable than PreF, the nAb titer induced by the antigenic epitopes on its surface is lower for PostF than for PreFl; furthermore, the vaccine designed on the basis of PostF may not protect humans from hRSV infection well, leading to the failure of vaccine development. In recent years, PreF has attracted considerable attention as a new immunogen. PreF can be expressed at the early stage of hRSV infection, and it is more likely to induce a broader-spectrum nAb response than PostF.

### 4.3 Differences in immune populations

At present, hRSV vaccines are under development for three age groups: infants and young children, pregnant women, and older adults. However, developing a vaccine applicable to all three groups simultaneously remains difficult because of the differences in the maturity of the immune system and the level of specific antibodies against hRSV among the three age groups. Infants and young children have a high risk of severe disease after hRSV infection and require early immunization to survive the hRSV epidemic season. However, the immune systems of infants and young children are not underdeveloped, and the immune response generated through natural infection with hRSV is susceptible to the level of maternal antibodies in their bodies (Murphy et al., 1986). As such, live-attenuated vaccines (LAVs) or vector vaccines may be used as much as possible in seronegative infants and young children, and vaccination with nonreplicating (inactivated or subunit) vaccines may lead to ERD development (Schneider-Ohrum et al., 2017). Currently, an effective protective measure for infants and young children is the immunization of pregnant women at the time of delivery. hRSV-nAbs produced by the mother can be transmitted through the placenta and have a half-life of 3–6 months in the infant, affording the infant protection against hRSV infection during this period (Ochola et al., 2009; Chu et al., 2014; Buchwald et al., 2020.). In contrast, older adults demonstrate immune senescence, as evidenced by a decrease in hRSV-specific nAb titers with age, and they may be repeatedly infected with hRSV (Walsh et al., 2004). Therefore, vaccines for older adults must provide maximized immunoprotection. In general, consensus on the optimal population for hRSV vaccination has not been reached thus far; moreover, broad-spectrum universal vaccines suitable for all major age groups remain unavailable. Therefore, further research determining the optimal vaccine platform to maximize efficacy is warranted.

## 5 hRSV vaccines under clinical trials

To mitigate the impact of hRSV infection on human health, the World Health Organization (WHO) declared hRSV vaccine development a priority (Sparrow et al., 2022). As shown in Table 2, More than 20 hRSV vaccine candidates are currently under clinical development worldwide; these vaccines are based on one of these five mechanisms: (i) live-attenuated, (ii) recombinant-vector, (iii) subunit, (iiii) particle-based, and (iiiii) mRNA (Figure 1B). And the strengths and limitations of each candidate are summarized in Table 3. Out of these, four of the potential candidates are in phase III clinical trials, of which three have been given the go-ahead for usage (GSK plc, 2023; Pfizer Inc, 2023; Moderna Inc, 2024).

**TABLE 2:** hRSV vaccine candidates under clinical development.

Candidate	Antigen	Adjuvant	Sponsor	Phase I trial	Phase II trial	Phase III trial	Results
**LAV**							
SP0125 (RSV LID/ΔM2-2/ 1030s, RSVt)	All viral proteins except M2-2	None	NIAID (Medi/Sanofi)	2016/07/15-2017/07/07; NCT02794870; 6–24-month-old children; 33 participants 2020/09/17-2023//04/13; NCT04491877; 6–18-month-old children; 259 participants 2022/02/23-2024/12/01; NCT04520659; hRSV-seronegative infants and 6–24-month-old children; 81 participants 2023/02/06-2025/05/21; NCT05687279; 6–23-month-old children; 80 participants	2020/09/17-2023//04/13; NCT04491877; 6–18-month-old children; 259 participants 2023/02/06-2025/05/21; NCT05687279; 6–23-month-old children; 80 participants	2024/02/06-2026//06/02; NCT06252285; 6–22-month-old children; 6300 participants 2024/05/13-2026//03/10; NCT06397768; 6/12-month-old children; 2226 participants	Phase I and II: SP0125 demonstrated a significant reaction (93%) after utilizing the high-dose formula twice, and there was only a slight variation between the low-dose and high-dose formulae
sRSV 276	All viral proteins except M2-2	None	NIAID (Sanofi)	2017/09/22-2020/10/01; NCT03227029; 6–24-month-old hRSV-seronegative children; 65 participants 2019/05/16-2024/04/30; NCT03916185; 6–24-month-old hRSV-seronegative children; 67 participants	2019/05/16-2024/04/30; NCT03916185; 6–24-month-old hRSV-seronegative children; 67 participants	–	96% of RSV 276 recipients were infected with vaccine; serum RSV-neutralizing titers and anti-RSV F IgG titers increased ≥ 4-fold in 92% of RSV/276 vaccinees
RSV ΔNS2/Δ1313/ I1314L	All viral proteins except NS2 and SH	None	NIAID (Medi/Sanofi)	2013/06/01-2025/10/01; NCT01893554; 12–59-month-old hRSV-seropositive children, 6–24-month-old hRSV-seronegative, and 4–6-month-old children; 88 participants 2017/09/22-2020/10/01; NCT03227029; 6–24-month-old hRSV-seronegative children; 65 participants 2019/05/16-2024/04/30; NCT03916185; 6–24-month-old hRSV-seronegative children; 67 participants	2019/05/16-2024/04/30; NCT03916185; 6–24-month-old hRSV-seronegative children; 67 participants	–	Phase I: 88% of the recipients were infected with vaccine; serum RSV-neutralizing titers and anti-RSV F IgG titers increased ≥ 4-fold in 60% of RSV ΔNS2/Δ1313/I1314L Phase I and II: In RSV-seronegative children, the 10^5^ PFU dose was overattenuated, but the 10^6^ PFU dose was well tolerated, infectious and immunogenic
RSV 6120/ΔNS2/ 1030s	All viral proteins except NS2	None	NIAID (Sanofi)	2017/10/13-2021/05/31; NCT03387137; 12–59-month-old hRSV-seropositive children and 6–24-month-old hRSV-seronegative children; 45 participants 2019/05/16-2024/04/30; NCT03916185; 6–24-month-old hRSV-seronegative children; 67 participants	2019/05/16-2024/04/30; NCT03916185; 6–24-month-old hRSV-seronegative children; 67 participants	–	Phase I and II: RSV/6120/ΔNS2/1030s infected 100% of RSV-seronegative vaccinees and was immunogenic and genetically stable; mild rhinorrhea was detected more frequently in vaccinees and LRI occurred in 1 vaccinee during a period when only vaccine virus was detected; following the RSV season, 5 of 16 vaccinees had ≥ 4-fold rises in RSV plaque-reduction neutralizing antibody titer (RSV-PRNT) with significantly higher titers than 4 of 10 placebo recipients with rises
RSV 6120/ ΔNS1	All viral proteins except NS1	None	NIAID (Sanofi)	2018/06/25-2024/09/30; NCT03596801; 12–59-month-old hRSV-seropositive children and 4–6- and 6–24-month-old hRSV-seronegative children; 75 participants	–	–	–
RSV 6120/F1/G2/ ΔNS1	All viral proteins except NS1	None	NIAID (Sanofi)	2018/06/25-2024/09/30; NCT03596801; 12–59-month-old hRSV-seropositive children and 4–6- and 6–24-month-old hRSV-seronegative children; 75 participants	–	–	–
MV-012-968	All viral proteins	None	Meissa Vaccines	2020/01/14-2020/08/27; NCT04227210; 18–40-year-old adults; 20 participants 2020/06/09-2021/05/07; NCT04444284; 15–59-month-old hRSV-seropositive children; 34 participants 2021/06/03-2023/10/01; NCT04909021; 6–36-month-old children; 63 participants	2020/12/29-2021/09/09; NCT04690335; 18–45-year-old adults; 60 participants	–	Phase I: Well tolerated, heavily attenuated, and induced an hRSV-specific mucosal IgA response in healthy seropositive participants; induced a robust serum nAb response in infants not previously exposed to hRSV; 100% of hRSV-naïve infants and toddlers responded to two doses of 10^7^ plaque-forming units
CodaVax-RSV	Codon deoptimized hRSV	None	Codagenix	2020/07/10-2021/05/26; NCT04295070; 18–49- and 50–75-year-old adults; 36 participants 2023/03/28-2024/06/05; NCT04919109; 6–24-month-old hRSV-seronegative children and 2–5-year-old hRSV-seropositive children; 51 participants	–	–	–
**Recombinant vector vaccine**							
BLB-201	F protein	None	Blue Lake Biotechnology	2022/07/20-2023/05/03; NCT05281263; 18–75-year-old adults; 30 participants 2023/03/09-2024/12/23; NCT05655182; 6–24-month-old hRSV-seropositive and -seronegative infants and 18–59-month-old children; 137 participants	2023/03/09-2024/12/23; NCT05655182; 6–24-month-old hRSV-seropositive and -seronegative infants and 18–59-month-old children; 137 participants	–	Phase I and II: Five participants receiving 10^7^ plaque-forming units had prominent increases in hRSV nAb responses at 4 weeks after vaccination, with 80% having a 3.6- to 57-fold increase in nAbs over baseline; induced hRSV-specific mucosal IgA and cellular immune responses
RSV/Flu-01E	F protein	None	Tatyana Zubkova	2023/05/10-2023/09/17; NCT05970744; 18–59- and ≥ 60-year-old adults; 60 participants	–	–	–
**Subunit vaccine**							
Arexvy (RSV PreF3 OA)	F protein	AS01/AS01E	GlaxoSmithKline	2019/09/25-2020/12/11; NCT04090658; 60–80-year-old adults; 40 participants	2019/01/21-2020/11/30; NCT03814590; 18–40- and 60–80-year-old adults; 1053 participants 2020/12/09-2021/10/25; NCT04657198; 60–80-year-old adults and a boost of participants from NCT03814590; 126 participants 2023/07/28-2025/08/13; NCT05921903; ≥ 18-year-old renal or lung transplant patients and ≥ 50-year-old healthy adults; 375 participants	2021/04/27-2022/02/08; NCT04841577; 60-year-old adults; 976 participants 2021/02/15-2024/05/25; NCT04732871; 60-year-old adults; 1720 participants 2021/05/25-2024/05/31; NCT04886596; 60-year-old adults; 26668 participants 2022/10/14-2023/07/17; NCT05568797; ≥ 65-year-old adults; 1045 participants 2022/10/20-2023/08/15; NCT05559476; ≥ 65-year-old adults; 1029 participants 2022/10/28-2024/02/14; NCT05590403; 50–59- and ≥ 60-year-old adults; 1576 participants	Phase I and II: Humoral and cellular immune responses for all vaccines; higher humoral response in older adults with higher dosage and higher cellular response with adjuvant Phase III: Statistically significant and clinically meaningful overall efficacy of 82.6% against hRSV-LRTD, 94.1% efficacy against severe hRSV-LRTD and 71.7% against hRSVALRI among older adults ≥ 60 years, defined as an RSV-associated LRTD episode preventing normal daily activities—consistent between hRSV subtypes A and B
Abrysvo	F protein	None or aluminum salts	Pfizer	–	2018/04/18-2020/12/28; NCT03529773; 18–49- and 50–85-year-old adults; 1235 participants 2018/06/05-2020/08/19; NCT03572062 65–85-year-old adults; 317 participants 2019/08/07-2021/09/30; NCT04032093; 18–49-year-old pregnant women; 1153 participants 2019/10/01-2019/12/11; NCT04071158; 18–49-year-old nonpregnant women; 713 participants	2020/06/17-2023/11/24; NCT04424316; 18–49-year-old pregnant women; 14750 participants 2021/08/31-2026/06/12; NCT05035212; ≥ 60-year-old adults; 38567 participants 2021/10/21-2022/04/04; NCT05096208; 18–49-year-old adults; 1028 participants 2022/04/13-2022/10/12; NCT05301322; ≥ 65-year-old adults; 1471 participants 2023/05/11-2024/03/18; NCT05842967; 18–59-year-old adults; 886 participants 2023/06/22-2024/02/29; NCT05900154; 2–17-year-old children with high hRSV infection risk; 128 participants	Phase I and II: Safe and well tolerated; immunization elicited 10–20-fold increases in nAb titers Phase III: Vaccine efficacy of 66.7% in participants with hRSV-associated LRTD with at least two signs or symptoms, 85.7% in participants with at least three signs or symptoms; participants also achieved at least a fourfold increase in serum nAb titers for hRSV subtypes A and B over 1 month
RSVpreF	F protein	None	Hvivo	–	2020/11/10-2021/08/16; NCT04785612; 18–50-year-old adults; 70 participants	–	Vaccine efficacy of 86.7% for symptomatic RSV infection confirmed by any detectable viral RNA over at least 2 consecutive days; the geometric mean factor increase from baseline in hRSV subtype A nAb titers 28 days after injection of 20.5 and 1.1 in the vaccine and placebo groups, respectively
SCB-1019	F protein	None	Clover Biopharmaceuticals	2023/12/13-2025/05; NCT06194318; 18–59- and 60–85-year-old adults; 60 participants	–	–	Significantly boost in hRSV subtypes A and B nAb titers to approximately 6,600 and 46000 IU/mL, respectively (6.4- and 12-fold increase, respectively)
BARS13 (ADV110)	G protein	AE011	Advaccine Biopharmaceuticals	2018/10/16-2019/08/02; NCT04851977; 18–45-year-old adults; 60 participants	2021/05/24-2024/03/31; NCT04681833; 60–80-year-old adults; 125 participants	–	Phase I: Generally good safety and tolerability profile; no significant difference in terms of adverse reaction severity or frequency between different dose groups Phase I: BARS13 vaccination increased IgG anti-RSV antibody levels in all cohorts.
VN-0200	VAGA-9001a	MABH-9002b	Daiichi Sankyo	2021/06/11-2021/12/16; NCT04914520; 20–50- and 65–80-year-old adults; 48 participants	2022/10/13-2024/02/15; NCT05547087; 60–80-year-old adults; 342 participants	–	–
**Particle-based vaccine**							
V-306	Conjugate (lipopeptide building block/universal T-helper epitope/palivizumab epitope mimetic)	Pam2Cys	Virometix	2020/09/07-2022/03/02; NCT04519073; 18–45-year-old nonpregnant women; 60 participants	–	–	Safe and well tolerated at all dose levels; at 50 and 150 μg, induced an increase in FsIIm-specific IgG titers, lasting at least 4 months
IVX-121	F protein	Aluminum salts	Icosavax	2021/07/19-2022/03/12; 2020-003633-38; 18–45- and 60–75-year-old adults; 220 participants	–	–	Favorable tolerability; high hRSV subtypes A and B nAb titers from the lowest dose tested; similarly robust responses in older and younger adults; six-month immunogenicity update showing durability of hRSV subtypes A and B nAb titers up to 180 days after vaccination, with geometric mean titers against hRSV subtype A through day 180 persisting at 64–98% of the GMTs at day 28 in older adults
IVX-A12	hRSV-F protein/ hMPV-F protein	MF59	Icosavax	2022/09/21-2024/01/24; NCT05664334; 60–75-year-old adults; 140 participants	2023/05/15-2024/09/30; NCT05903183; 60–85-year-old adults; 264 participants	–	Phase I: Robust immune responses to both hRSV and hMPV in older adults; higher postvaccination levels of nAbs against hRSV subtypes A and B in this study than with IVX-121 alone
**mRNA vaccine**							
mRESVIA (mRNA-1345)	F protein	LNP	Moderna	2020/09/30-2024/06/29; NCT04528719; 18–49- and 65–80-year-old older adults and 12–60-month-old hRSV-seropositive children; 651 participants 2023/02/15-2026/07/30; NCT05743881; 5–8- and 8–24-month-old children; 210 participants	2021/11/17-2025/08/25; NCT05127434; ≥ 60-year-old adults; 36814 participants 2023/10/24-2024/07/19; NCT06097299; 2–5- and 5–18-year-old children; 340 participants 2023/11/15-2026/02/18; NCT06143046; ≥ 18- to < 40-year-old pregnant women; 360 participants	2021/11/17-2025/08/25; NCT05127434; ≥ 60-year-old adults; 36814 participants 2022/04/01-2025/01/06; NCT05330975; ≥ 50-year-old adults and older adults; 3800 participants 2023/10/06-2025/12/29; NCT06067230; 18–60-year-old adults with at least one listed condition (coronary artery disease, congestive heart failure, CLD, stable type 1 or 2 diabetes) and ≥ 18-year-old solid organ transplant recipients; 1150 participants	Phase I: Well-tolerated at all dose levels; a single injection boosted hRSV subtypes A and B nAb and preF binding antibody concentrations at 1 month after injection, without apparent dose response; antibody levels remained higher than baseline through 6 months Phase II and III: 83.7% vaccine efficacy against hRSV-associated LRTD with at least two signs or symptoms, 82.4% against the disease with at least three signs or symptoms, and 68.4% against hRSV-associated acute respiratory disease; protection against both hRSV subtypes A and B, generally consistent across subgroups defined according to age and coexisting conditions
SP0256	F protein	LNP	Sanofi	2022/11/17-2025/04/29; NCT05639894; 18–50- and ≥ 60-year-old adults; 790 participants	2022/11/17-2025/04/29; NCT05639894; 18–50- and ≥ 60-year-old adults; 790 participants	–	Phase I and II: SP0256 was well tolerated and significantly boosted hRSV nAb responses

–means not applicable or not available.

**TABLE 3:** The candidates’ advantages and shortages.

Candidate		Advantage	Shortage
**LAV**			
Vaccines knocking out the M2-2 gene	SP0125 (RSV LID/ΔM2-2/1030s, RSVt)	SP0125 is a nasal spray attenuated live vaccine suitable for children of all ages. It provides protection against RSV infection during the second epidemic season.	–
	RSV 276	RSV 276 demonstrated excellent infectivity and was well tolerated, showing strong immunogenicity and priming robust anamnestic responses.	RSV 276 induced an excess of mild cough.
Vaccines knocking out the NS2 gene	RSV ΔNS2/Δ1313/I1314L	RSV ΔNS2/Δ1313/I1314L better balances vaccine stability and toxicity compared to other candidates with NS2 deletions or non-stabilized *ts* mutations.	All-cause medically attended acute respiratory illness (MAARI) occurred frequently among participants, and several other viruses were detected. These may have impacted RSV replication or immunogenicity.
	RSV 6120/ΔNS2/1030s	RSV/6120/ΔNS2/1030s was less temperature sensitive and less restricted in experimental animals than RSV/ΔNS2/Δ1313/I1314L.	An increase in mild rhinorrhea and cough or pediatric lower respiratory illness (LRI) would be concerning.
Others	MV-012-968	MV-012-968 is an intranasal spray preparation that can induce both serum IgG and nasal mucosa IgA responses. It demonstrated superior tolerability compared to other LAVs.	–
	CodaVax-RSV	CodaVax RSV exhibits genetic stability and safety while simultaneously inducing both cellular and humoral immune responses.	–
**Recombinant vector vaccine**			
PIV5 vector vaccine	BLB-201	The intranasal vaccine BLB201 is safety and ability to induce antibody and cell-mediated immune responses.	–
**Subunit vaccine**			
PreF subunit vaccines	Arexvy	Arexvy is the world’s first PreF subunit vaccine designed specifically to safeguard older adults over 60 years.	Arexvy does not provide adequate protection to frail individuals and older adults over 80 years. And it have led to Guillain Barre syndrome (GBS) development in patients in the vaccine group.
	Abrysvo	Abrysvo is a bivalent vaccine consists of PreF from hRSV A and B, and has been approved to be the world’s first hRSV maternal vaccine that can protect both infants and old adults.	Abrysvo offers protection that is not significantly superior to that provided by monoclonal antibodies in infants. And it have led to GBS development in patients in the vaccine group.
	SCB-1019	Clover’s PreF antigens in SCB-1019 are in the stabilized prefusion and trimeric form. SCB-1019’s preliminary immunogenicity data across both hRSV A and B neutralization appear to be in-line or potentially favorable compared to other PreF subunit vaccines.	–
G protein subunit vaccines	BARS13 (ADV110)	BARS13 is a bivalent vaccine developed for both subtypes G1 and G2, and has been noted to demonstrate safety, tolerability, and immunogenicity in 18–80 adults.	–
**Particle-based vaccine**			
VLPs vaccines	V-306	V-306 displays multiple RSV F site II protein mimetics as the antigenic epitope by the synthetic virus-like particle (SVLP) platform technology.	No increase in anti-F protein-specific and IgG RSV-neutralizing antibody titers was observed due to past natural infections.
	IVX-A12	IVX-A12 is a potential combination vaccine candidate containing VLPs that incorporate stabilized PreFs from hRSV and hMPV viruses. The FDA has granted IVX-A12 Fast Track designation in older adults over 60 years.	–
**Nucleic acid vaccine**			
mRNA vaccines	mRESVIA (mRNA-1345)	mRNA-1345 offers rapid production capacity and strong immunogenicity, effectively stimulating immune responses against hRSV. It was approved to be the world’s first hRSV mRNA vaccine.	mRNA vaccines may face storage and transportation challenges, such as the need for low-temperature preservation. Additionally, further long-term research is required to assess the ongoing safety and effectiveness.
	SP0256	SP0256 is an RSV-hMPV-PIV triple mRNA vaccine mainly used for the elderly.	–

–means not applicable or not available.

### 5.1 LAVs

To screen for temperature-sensitive attenuated vaccines, the early stage of LAV development mostly involves conventional viral attenuation techniques such as cold passaging and chemical mutagenesis. In recent years, reverse genetics methods have been used to insert predefined mutated complementary DNAs, obtained through cloning, into live hRSV, which provides attenuated, more immunogenic live hRSV strains. In addition, LAVs administered via nasal drops mimic natural human infections, effectively activating local mucosal immunity in the respiratory tract and systemic intrinsic, humoral, and cellular immunity in infants and young children; this method is not affected by maternal antibodies and does not cause ERDs, making it suitable for infants and young children aged 6–24 months and seronegative for hRSV (Mazur et al., 2018; Teng et al., 2020). At present, the main approach used to develop LAVs involves knocking out or modifying protein genes crucial for the regulation of synthesis of hRSV RNAs (e.g., those encoding the nonstructural proteins M2-2 and NS2), which eventually leads to viral replication restriction. In some LAV development approaches, all hRSV viral proteins are preserved and the protein sequences are optimized through codon deoptimization, resulting in reducing translation of target protein and thus restricting viral replication (Coleman et al., 2008). However, these methods are associated with a risk of virulence restoration, and excessive reduction of viral replication can lead to loss of immunogenicity. Thus, a balance of safety and immunogenicity is essential during the LAV development process.

#### 5.1.1 Vaccines knocking out the M2-2 gene

M2-2 gene knockout can enhance the immunogenicity of LAVs, inhibiting viral RNA replication and promoting F and G protein expression on hRSV surfaces. The LAV candidate SP0125 (i.e., LID/ΔM2-2/1030s, RSVt), based on LID/ΔM2-2 (McFarland et al., 2018), was developed by knocking out the M2-2 gene and inserting the temperature-sensitive phenotypic point mutation 1030s. The results of a Phase I clinical trial demonstrated that SP0125 showed high genetic stability in hRSV-seronegative infants aged 6–24 months. In 90% of these infants, the vaccine induced both nAbs and anti-hRSV F IgGs, which were sustained until the subsequent hRSV epidemic season (McFarland et al., 2020). Phase I/II clinical trials revealed that two doses of SP0125 were as well-tolerated as a placebo and successfully induced antibody responses in 93% of participants. It has advanced to Phase III clinical trials and is currently the fastest-progressing LAV.

#### 5.1.2 Vaccines knocking out the NS2 gene

NS2 gene knockout reduces hRSV’s replication ability, stimulates interferon production, and enhances intrinsic immunity (Sedeyn et al., 2019). The RSV ΔNS2/Δ1313/I1314L, with NS2 deletion and temperature sensitivity, has entered phase II clinical trials (Karron et al., 2020). Phase I clinical trials demonstrated that this vaccine candidate led to sufficient attenuation and genetic stability *in vivo* (Cunningham et al., 2022). However, it may be over-attenuated when tested in larger clinical trials, prompting the development of RSV/6120/ΔNS2/1030s as an alternative. This candidate also features the NS2 deletion but has a slightly less restricted replication profile. As a result, it is expected to be less replication-restricted and more immunogenic than RSV ΔNS2/Δ1313/I1314L in young children. Phase I/II clinical trials showed that RSV/6120/ΔNS2/1030s is both immunogenic and genetically stable in hRSV-seronegative children, although the incidence of rhinorrhea, cough, and pediatric lower respiratory illness (LRI) in vaccinees warrants attention (Karron et al., 2024).

#### 5.1.3 Other LAVs

MV-012-968, created by Meissa Vaccines using its exclusive codon deoptimization platform AttenuBlock, is an LAV devoid of adjuvant elements. During phase I clinical trials, this vaccine demonstrated pronounced attenuation and favorable tolerability, triggering robust hRSV-specific mucosal IgA responses in both adult and child participants with hRSV infection history along with detectable hRSV antibodies in their blood. In hRSV-naive infants, MV-012-968 also stimulated strong serum nAb responses. Moreover, compared with other LAV candidates, MV-012-968 demonstrated superior tolerability (Karron et al., 2021; Meissa Vaccines Inc, 2023).

Another potential intranasal hRSV LAV candidate is CodaVax-RSV, in which gene editing techniques are employed for codon deoptimization so as to protect against wildtype regression. Preclinical data indicated that CodaVax-RSV is significantly less virulent than wildtype hRSV; furthermore, it elicits both nAb and cellular immune responses and affords effective protection against wildtype infections with a high safety profile. This vaccine has received the US FDA’s fast-track designation and is presently undergoing phase I clinical trials in healthy children (Farmingdale, 2023).

### 5.2 Recombinant vector vaccines

Recombinant vector vaccines are designed to express hRSV target protein antigens by using related LAVs as vectors. These vector-based vaccines have demonstrated a favorable safety profile and can effectively stimulate antigen presentation to elicit adaptive immune responses. Various viral vectors have been employed to create hRSV vaccines such as adenoviral (AdV) vectors, modified vaccine Ankara (MVA) vectors as well as parainfluenza virus 5 (PIV5) vectors. However, the termination of multiple hRSV vector vaccines after entering the clinical stage has prevented the use of the vector vaccine platform.

The candidate vaccine BLB-201 includes an attenuated strain of PIV5 as a viral vector, which expresses hRSV F protein, stimulating immune protection via intranasal administration that simulates natural infection. Phase I clinical trials of BLB-201 are currently underway in 33-75-year-old healthy adults who are hRSV seropositive. Initial findings have demonstrated the safety of BLB-201. Moreover, 48% of the participants have demonstrated specific IgA responses to hRSV, with the highest level being observed in clinical trials on healthy adults who were administered hRSV vaccines intranasally (Spearman et al., 2023). A Phase I/IIa clinical trial revealed a notable surge in nAbs 4 weeks after vaccination in five individuals administered with a high dose of BLB-201. Among these individuals, 80% demonstrated a substantial increase in nAb levels, ranging from 3.6- to 57-fold compared with the baseline (Athens and San, 2024). The US FDA had granted the fast-track designation to BLB-201 for the prevention of hRSV-related LRTD in older adults aged ≥ 60 years and children aged ≤ 2 years.

### 5.3 Subunit vaccines

Due to the low antigenic components and reduced immunogenicity, subunit vaccines typically require the inclusion of adjuvants or administration of multiple doses to elicit a sustained immune response. These vaccines primarily stimulate protective B-cell and CD4^+^T-cell production (Schneider-Ohrum et al., 2019). However, when administered to seronegative infants and young children, they may lead to ERD development. Therefore, subunit vaccines are being developed mainly for pregnant women and older adults. Currently, five hRSV subunit vaccines are under development (Crank et al., 2019); of them, three include PreF as the antigen, and two have been approved for marketing.

#### 5.3.1 PreF subunit vaccines

Among all hRSV subunit vaccines, most progress has been achieved with those based on PreF. The levels of nAbs produced in response to antigenic epitopes on the hRSV surface are closely associated with their neutralizing activity of the subunit vaccines. Furthermore, nAbs induced after natural hRSV infection primarily originate from the specific antigenic epitope site Ø, located on top of PreF (Magro et al., 2012). Hence, to ensure hRSV subunit protein vaccine stability, a protein structure maintaining PreF conformation must be designed (Crank et al., 2019).

On May 3, 2023, Arexvy (i.e., RSVPreF3 OA), a monovalent recombinant subunit vaccine from GSK plc (2023), was granted licensure by the US FDA. In this vaccine, GSK’s proprietary adjuvant AS01E is combined with the recombinant protein RSV PreF; it became the world’s first vaccine designed specifically to safeguard older adults aged ≥ 60 years.

Owing to thermal instability, PreF can conform to PostF during infection or purification, which results in the loss of neutralizing epitopes. As such, Arexvy employs a cavity-filling mutation and AS01 adjuvant strategy to enhance nAb production and CD4^+^T-cell activation. In phase III clinical trials, the vaccine demonstrated an overall efficacy of 82.6%, with a remarkable efficacy of 94.1% against LRTD and consistent prophylactic efficacy against hRSV subtypes A and B (Papi et al., 2023).

In phase III clinical trials, Abrysvo—an adjuvant-free bivalent vaccine candidate developed by Pfizer Inc (2023), consisting of PreF from subtypes A and B—demonstrated an overall efficacy rate of 85.7% in older adults aged ≥ 60 years. The vaccine effectively prevented two or more symptomatic hRSV-LRTD infections in these older adults (Walsh et al., 2023). Neonatal administration of Abrysvo through maternal vaccination yielded a protection efficiency of 81.8% within 90 days of birth and 69.4% effectiveness at 6 months after birth (Kampmann et al., 2023). On August 21, 2023, the US FDA-approved Abrysvo for active immunization of pregnant women between the gestational ages of 32 and 36 weeks so as to prevent hRSV-LRTD infections in newborns. Abrysvo is currently the only approved maternal hRSV vaccine.

Clinical trials of many hRSV subunit vaccines other than Arexvy and Abrysvo are also underway. A few of these vaccines have entered the clinical stage in China. SCB-1019 is a bivalent PreF trimeric subunit vaccine developed using Clover’s proprietary Trimer-Tag vaccine technology platform, which includes PreF from both hRSV subtypes A and B. In the first batch of young adults in the phase I clinical trial, SCB-1019 significantly increased the neutralization titers of hRSV subtypes A and B by approximately 6.4- and 11.8-fold, respectively (Clover Biopharmaceuticals, 2024).

#### 5.3.2 G protein subunit vaccines

In contrast to hRSV vaccines with F protein as the immunogen, BARS13 (i.e., ADV110) targets G protein; this vaccine has advanced to the phase II clinical trial stage in Australia. This unique approach not only prevents the virus from infecting cells but also prevents the virus from spreading through cell-cell fusion infections. Furthermore, a bivalent vaccine is currently being developed for both subtypes G1 and G2. A challenge associated with G protein vaccine development is the overactivation of cellular immunity, which leads to lung inflammation and mutation. To address it, a proprietary AE011 adjuvant has been added to BARS13, which inhibits the overactivation of the immune response, increases nAb titer, and enhances safety. In phase I clinical trials, BARS13 has been noted to demonstrate safety, tolerability, and immunogenicity in healthy adults aged 18–45 years (Cheng et al., 2023). Moreover, phase II clinical trial data from a large sample size have reinforced these positive attributes of BARS13 in older adults aged 60–80 years (Lunan et al., 2023).

### 5.4 Particle-based vaccines

Particle-based vaccines demonstrate the immunogenicity of hRSV antigenic proteins through particle assembly; the addition of adjuvants can further enhance their immunogenicity and target antigen presentation (Jeong and Seong, 2017). However, because of the ineffectiveness of the vaccine candidate ResVax, research on particulate vaccines against hRSV has decelerated significantly (August et al., 2017): only three particulate vaccine candidates are presently in the clinical trial stage.

The vaccine candidate V-306 is based on synthetic virus-like particles (SVLPs) presenting epitopes of the monoclonal nAb palivizumab in the region of the F protein epitope site II. The safety and immunogenicity of V-306 have been assessed in cotton rats, mice, and rabbits (Zuniga et al., 2021). This vaccine candidate is designed to augment preexisting immunity in pregnant women and older adults, and it is currently being evaluated in phase I clinical trials on healthy women.

IVX-121, developed by Icosavax, uses a platform technology that enables self-assembling SVLPs to deliver 20 stable trimeric PreFs. Relevant phase I/Ib clinical trial data have demonstrated that IVX-121 induces higher, longer-lasting nAb titers than the DS-Cav1 antigen, even when used in similar or lower antigen doses (Marcandalli et al., 2019). However, Icosavax does not intend to market IVX-121 as an hRSV vaccine candidate; instead, they have proposed IVX-A12, a bivalent vaccine consisting of the human metapneumovirus (hMPV) vaccine candidates IVX-121 and the IVX-241. Phase II clinical trials have demonstrated robust immune responses and favorable tolerability for IVX-A12, with significant hRSV and hMPV antibody responses. This particulate vaccine enables dense, multivalent presentation of antigens in a manner closely resembling the viral structure, inducing stronger, longer-lasting immune responses than traditional soluble antigens. The US FDA has granted the fast-track designation to IVX-A12 for the prevention of hRSV infections in individuals aged ≥ 60 years.

### 5.5 Nucleic acid vaccines

As third-generation vaccines, nucleic acid vaccines demonstrated both safety and efficiency throughout the COVID-19 pandemic (Corbett et al., 2020). mRNA vaccines have emerged as a novel alternative to traditional vaccines in hRSV vaccine development and production. Moderna has led relevant research and developed several hRSV mRNA vaccines. Although mRNA-1777 demonstrated favorable safety and humoral immune responses during phase I clinical trials, its further development has been terminated. This may be because after sequence optimization, compared with mRNA-1777, mRNA-1172 displayed superior efficacy in animal models. mRNA-1345 was designed for children based on mRNA-1772 with sequence enhancements to encode stable PreF by using the same lipid nanoparticle (LNP) delivery system as the COVID-19 vaccine SpikeVax. mRNA-1345 can induce both nAb and T-cell responses. Notably, nAb titers elicited by mRNA-1345 were approximately eightfold those elicited by mRNA-1777. In a pivotal phase III clinical trial, mRNA-1345 met the primary efficacy endpoint: 83.7% vaccine efficacy when two or more hRSV-LRTD symptoms were present and 82.4% vaccine efficacy when three or more hRSV-LRTD symptoms were noted; moreover, mRNA-1345 displayed a good safety profile and tolerability (Moderna Inc, 2023). As anticipated, mRNA-1345 (i.e., mRESVIA) was approved for marketing by the US FDA on 31 May 2024, making it the world’s first hRSV mRNA vaccine designed to prevent hRSV in older adults aged ≥ 60 years.

## 6 The monoclonal antibodies treatments

When it comes to controlling hRSV infection and disease, monoclonal antibodies (mAbs) treatments are just as crucial as vaccinations. Advancements in comprehension of the structure and immunogenicity of hRSV F protein have led to the development of next-generation antibodies that specifically target highly neutralization-sensitive epitopes on PreF. Nirsevimab (i.e., MEDI-8897) is a recombinant human IgG1 kappa mAb that specifically targets the site Ø epitope unique to PreF and simultaneously neutralizes multiple hRSV A/B subtypes. It features a YTE mutation in the Fc region to prolong its half-life to 63–73 days *in vivo* (Hammitt et al., 2022). For the duration of the hRSV epidemic season, a single injection of Nirsevimab can provide protection against hRSV infection for up to five months (Domachowske et al., 2018; Griffin et al., 2020). In the phase III trial (MELODY), Nirsevimab shows 74.5% efficacy against medically attended LRTD, 76.8% against LRTD-related hospitalizations and 78.6% against very severe medically attended LRTD in term and late-preterm infants (Muller et al., 2023). On 17 July 2023, the FDA approved Nirsevimab for marketing, making it the first long-acting preventive mAb treatment for all infants against hRSV.

## 7 Discussion

hRSV infection imposes a substantial disease burden on infants and older adults. Consequently, the development of a safe, efficacious hRSV vaccine is essential for preventing hRSV infection, mitigating severe symptoms, and reducing mortality. With a deeper understanding of hRSV’s molecular structure and immune response in humans, hRSV vaccine development has progressed, with a notable transition from empirical to rational vaccine design. The development of different hRSV vaccine types has encountered various hindrances because the creation of a safe, effective hRSV vaccine is associated with considerable challenges. However, these failures have laid the groundwork for the eventual success of such a vaccine. In the more than six decades history of hRSV vaccine development, PostF has been selected as the immunogen in numerous candidates. Despite being in a stable structure, it is unable to effectively prevent hRSV by inducing high-level nAbs. The current phase of hRSV vaccine development, based on previous results, involves predominant use of PreF as the antigen. Ensuring F protein’s stability in the PreF conformation has emerged as a pivotal consideration in vaccine formulation. At present, most research emphasizes eliciting potent nAbs after vaccination, as well as mitigating non-nAb production and ERD responses, in recipients. An ideal hRSV vaccine should be able to induce highly efficient cellular immune responses based on nAbs and type 1 T helper cells and provide protection to individuals across all age groups that require vaccination.

Currently, > 20 candidate vaccines have entered the clinical trial phase, targeting different high-risk populations vulnerable to hRSV infection. Despite having some levels of maternal immunity, infants aged ≤ 6 months continue to have a high risk of LRTD after hRSV infection. Therefore, maternal vaccination remains a highly effective strategy for hRSV prevention and ERD mitigation in infants. Bolstering maternal hRSV antibody levels at least 3 months before delivery can facilitate antibody transfer via the placenta. Abrysvo, the first US FDA-approved maternal vaccine, confers extensive protection to expectant mothers and provides an alternative to parents who hesitate to vaccinate their infants. With the advent of mAbs and maternal vaccines for infant protection, the focus has shifted to how to balance the use of these two preventive strategies. Nirsevimab can safeguard both preterm and full-term infants, offering longer-lasting protection compared to maternal vaccines and allowing for flexible application during hRSV seasonal fluctuations. mAb can be administered without maternal immunity, and combining both approaches offers an additional layer of protection. Maternal vaccines can also be fallback options, preventing viral resistance to mAbs and reducing high costs associated with the production of biologics; nevertheless, further improvement in maternal vaccines is warranted. However, Abrysvo provides protection that is not significantly better than that provided by the relevant monoclonal antibody in infants. In addition, Arexvy does not provide adequate protection to frail individuals and older adults aged ≥ 80 years (with only 14 and 34% efficacy, respectively). However, both GSK’s and Pfizer’s hRSV vaccines have led to Guillain Barre syndrome (GBS) development in patients in the vaccine group during phase III clinical trials (*n* = 1 and 2, respectively). Although the number of GBS cases has been limited in these trials, the safety concerns associated with these vaccines warrant attention.

The conventional monovalent hRSV vaccine technology may not fully meet all the demands, which makes the parallel development of multiple technology-based approaches a more practical choice. The use of combination vaccines may significantly reduce the number of vaccine doses required and increase vaccination willingness among the relevant population. Combination vaccines that simultaneously target multiple viruses are emerging mRNA vaccine modalities. Moderna, a United States-based company, plans to integrate mRNA-1345 with human metapneumovirus/parainfluenza virus 3 mRNA-1653 to create a single formulation for childhood vaccination against three pathogens. The company aims to offer a booster trivalent vaccine that can target SARS-CoV-2, influenza, and hRSV, thereby eliciting protection against multiple pathogens through a single vaccination (Qiu et al., 2022). However, mRNA vaccines are relatively new modalities aimed at use in humans; as such, mRNA vaccine development remains associated with several challenges, requiring further optimization, such as *in vitro* stability, delivery system optimization, protein translation efficiency, and nucleic acid impurity removal, during the production process. Clearly, the research of vaccines against infectious diseases has benefited from the multivalent vaccine development approach, and the progress made in the development of hRSV vaccines is leading to more promising applications.
